# Toward a Customized Program to Promote Physical Activity by Analyzing Exercise Types in Adolescent, Adult, and Elderly Koreans

**DOI:** 10.1515/hukin-2015-0027

**Published:** 2015-04-07

**Authors:** Sangwoo In, Wi-Young So

**Affiliations:** 1 Department of Physical Education, Myongji University, Gyeonggi, Republic of Korea.; 2 College of Humanities and Arts, Sports and Health Care Major, Korea National University of Transportation, Chungju-si, Republic of Korea.

**Keywords:** physical health state, exercise frequency, exercise intensity, exercise time, exercise duration

## Abstract

The purpose of this study was to examine the relationship between the perceived physical health status of Korean adolescents, adults, and elderly adults and their frequency, intensity, time, and duration of exercise. In 2012, 1,144 adolescents (under 18 years old), 6,474 adults (19–64 years old), and 1,382 elderly adults (over 65 years old) participated in the Korean Survey on Citizens’ Sports Participation Project (N = 9,000). The association between self-reported health status and exercise was assessed using multivariate logistic regression analyses, controlling for sex and age. The study found that the health status of adolescents showed little or no association with the frequency, intensity, time, or duration of exercise. However, the health status of adults and elderly Koreans was associated with the frequency, intensity, time, and duration of exercise. The physical condition and health status of adolescents was better than that of adults and the elderly, many of whom had declining health. Our findings show the need for exercise-promotion programs customized for particular age groups. The limitations and strengths of the study are discussed, as well as the implications for future research and managerial applications for promoting exercise in each age group.

## Introduction

Participating in daily sports activities is a crucial factor in the successful management of one’s own health (Sandvik et al., 1993). Regular exercise such as that found in sports, can reduce obesity, which is a potential cause of cardiovascular diseases, diabetes, musculoskeletal disorders, some cancers, and death (Apor, 2011; Colberg et al., 2010; Dishman et al., 2004). Obesity due to a lack of exercise has become a major social and public health problem worldwide (World Health Organization, 2013a; World Health Organization, 2013b).

Physical activity is related to the reduction of risk factors for certain diseases (Colberg et al., 2010; Dishman et al., 2004). Thus, researchers and healthcare providers view regular exercise as a major contributor to risk reduction for coronary heart disease, stroke, diabetes, some cancers, and osteoporosis (Hagen et al., 2012; Penedo and Dahn, 2005; Schmitz et al., 2004). Regular exercise has positive effects on the physical and psychological health status of individuals. The elements of exercise programs may be classified into the following categories: frequency, intensity, time, and duration (Scott and Edward, 2012). However, there are no published studies on the ways in which frequency, intensity, time, and duration of exercise affect an individual’s physical health status. Although physical activity is unrelated to health benefits in youth (Bauman et al., 2002; Van Der Horst et al., 2007), the belief that physical activities offer health benefits may be the initial factor that stimulates youths’ involvement in them (Giles-Cortia and Donovan, 2002). The feelings of enjoyment and well-being may be strong factors in the motivation to continue participation in physical activities (Aaltonen et al., 2012). The individual’s perception of his or her health status may influence long-term sustenance of exercise. Perceptions, motivation, and long-term participation may vary according to age group.

The Korean sports industry has grown rapidly since the 1990s, but participation rates in physical activity have significantly decreased (average difference of 36.67%, from 1991 to 2012) for the last 20 years (Korea Ministry of Culture, Sports and Tourism, 2012). The government, school authorities, policy makers, and many researchers have tried to find suitable programs to promote physical activity and to increase the participation rates for health promotion, by stressing a healthier lifestyle. In order to meet the needs for physical activity, we need to review the relationship between perceptions of health and types of exercise among different age groups. The motivation and need for participation in physical activity may be different for each age group and understanding of each age group should help in designing physical activity programs that enhance continuous participation.

Therefore, the purpose of the present study was to explore the relationship between the perceived physical health status of Korean adolescents, adults, and elderly adults and their frequency, intensity, time, and duration of exercise. By gaining better understanding of the factors influencing this relationship, we will be able to design a customized program to promote physical activity among the Korean people that is appropriate for each age group.

## Material and Methods

### Participants

This study used data from the nationwide Korean Survey on Citizens’ Sports Participation, conducted by the Korean Ministry of Culture, Sports and Tourism. A total of 1,144 adolescents (under 18 years old), 6,474 adults (19–64 years old), and 1,382 elderly (over 65 years old) participated in the survey (N = 9,000) in 2012. The sampling method used the square root of the proportional allocation design within clustering and stratifications, such that the survey sampled all of South Korea. The data were collected and analyzed from a nationally representative sample of participants.

The details of the data collection procedure are described elsewhere (Korean Ministry of Culture, Sports and Tourism, 2012). As the survey did not collect private information, such as name, social security number, or home address, ethical approval was not waived. All study procedures were approved by the Korean Ministry of Culture, Sports and Tourism on 09-09-2013. The characteristics of participants are shown in Table 1.

### Dependent variables

Physical health status was evaluated in each participant by asking the following question: “[Q1] Which of the following do you think best describes your general physical health status?” Participants chose 1 of 5 responses: [1] very unhealthy, [2] quite unhealthy, [3] so-so, [4] quite healthy, and [5] very healthy. To perform multivariate logistic regression, the participants were divided into 2 groups: (1) so-so and below (reference group; responses [1–3]), and (2) quite and very healthy (responses [4–5]).

### Independent variables

Exercise frequency was measured using the following question: “Recently, how many days did you do over 30 minutes of exercise other than walking in your leisure time?” The response options were [1] none, [2] two or three times per month, [3] once per week, [4] two times per week, [5] three times per week, [6] 4 times per week, [7] 5 times per week, [8] 6 times per week, and [9] every day.

Exercise time was measured using the following question: “How many minutes did you do exercise?” The response options were [1] none, [2] under 59 minutes, [3] 60–119 minutes, [4] 120–179 minutes, and [5] over 180 minutes.

Exercise duration was measured using the following question: “How long have you participated in exercise?” The response options were [1] none, [2] under 23 months, [3] 24–47 months, [4] 48–71 months, [5] 72–95 months, and [6] over 96 months.

Exercise intensity was measured using the following question: “At which level of intensity do you exercise?” The response options were [1] none, [2] low intensity (a degree in which you could sing during exercise), [3] moderate intensity (a degree in which you could have a conversation with a person during exercise), and [4] vigorous intensity (a degree in which it is difficult to breath well during exercise).

### Covariates

The covariates in this study were sex (male or female) and age group (< 18), (19–64), or (> 64). The participants’ ages, as defined by the Korean Survey on Citizens’ Sports Participation, were used without modifications.

### Statistical analysis

Multivariate logistic regression analyses were conducted to determine whether physical health status was related to the frequency, intensity, time, and duration of exercise, adjusting for the covariates sex and age for each group (adolescents, adults, and elderly). The analyses were performed using SPSS version 18.0 (Chicago, IL, USA) and statistical significance was set at p < 0.05. The results of this study are presented as means ± standard deviations.

## Results

The results of the multivariate logistic regression analyses of physical health status in relation to frequency, intensity, time, and duration of exercise in adolescent, adult, and elderly Koreans are shown in Table 2. There was no significant association of exercise frequency with the physical health status of adolescents (Table 2). All levels of exercise frequency (except for 2–3 times per month) were significantly associated with better physical health among adults, although this pattern did not appear to show a linear trend. Similarly, all levels of exercise greater than once per week were associated with better physical health in the elderly, although no linear trend was apparent. Nevertheless, adults and elderly who exercised 6 times a week reported the highest physical health status.

The amount of time adolescents devoted to exercise had a marginally significant association with health status for exercise periods of less than 2 hours. The amount of time adults devoted to exercise was significantly associated with better health for periods of 60 min or longer, with health status increasing with the amount of exercise. All levels of exercise were associated with significantly better levels of health among the elderly, with health status improving with the amount of exercise. The odds ratios for the elderly participants tended to be higher than those for adults at all exercise times.

Exercise duration also had little effect on the physical health status of adolescents. Only adolescents who exercised for less than 2 years reported significantly higher health status than the reference group. All exercise durations of 2 years or more were significantly associated with better health status in adult and elderly participants. The odds ratios for the elderly participants tended to be higher than those for adults at most exercise durations.

Only the highest level of exercise intensity was significantly associated with better health among adolescents. Moderate and high-intensity exercises were significantly associated with better physical health among adults, and low and moderate-intensity exercises were significantly associated with better physical health among the elderly.

## Discussion

The purpose of this study was to examine whether the physical health status of adolescent, adult, and elderly Koreans is related to the frequency, intensity, time, and duration of exercise. This study found that, in general, the physical health status of adolescents showed little or no association with the frequency, intensity, time, and duration of exercise. However, the health status of adults and elderly Koreans was associated with the frequency, intensity, time, and duration of exercise.

Adolescence is a period in which rapid physical and psychological growth is affected by hormones, such as dopamine and growth hormone, culminating in sexual maturity (Christie and Viner, 2005). Our findings for adolescents may be explained from a motivational perspective. Adolescents value the hedonic aspects (e.g., enjoyment and fun) of exercise more than young adults and elderly people do. Furthermore, they do not recognize the necessity of enhancing their exercise participation due to their confidence in their health condition. Thus, their perception of their health status may not have encouraged them to put more effort into exercise.

From a managerial viewpoint, the development of exercise programs having a health-related educational purpose should be effective in leading adolescents to increase their exercise participation rate. Their increase in participation should lead to improvements in their physical health status. Considering the results of physical activity in advancing age, elapsed time is crucial for present and future sports participation (Brodersen et al., 2007). Early intervention should focus not only on the healthy benefits of exercise but also on other social benefits, which can be adapted into the design of physical activity programs.

Unlike adolescents, adults and the elderly are in a period of physical decline. Given the influence of declining physical activity, it appears that adults and the elderly recognize the necessity of improving their physical health (e.g., muscle strength). For these two age groups, there was an association between health status and exercise intensity.

From a managerial perspective, this finding provides insights into the two groups. First, the age range of the adults is broad; hence, there is a need to develop tailored programs for specific age groups, as adults have different physical conditions compared to the more homogeneous group of adolescents. Second, physical activity managers should make an effort to provide safe exercise programs to prevent injuries in the elderly who may otherwise decline to attend, as they age. Finally, to improve the perception of the physical health of adults and the elderly, all four exercise variables need to be considered. Additional well-designed studies should be performed in the future to determine the separate effects of each of the four variables. Furthermore, complementary strategies that are linked with frequency, intensity, time, and duration of exercise (Giles-Cortia and Donovan, 2002), or exercise settings and the level of exercise, should be included in physical activity programs.

There are two major limitations of this study. First, physical health status was assessed through self-report and not objectively measured. Therefore, objective measures of health status should be included in the research designs of future studies. Second, owing to the cross-sectional retrospective cohort study, causal relationships could not be determined. However, this study investigated a representative sample of South Korea with 9,000 participants. The large sample size facilitates generalization of the study’s findings.

## Conclusion

We conclude that, in general, physical health status is not significantly related to the frequency, intensity, time, and duration of exercise in adolescents, but such relationships are present and important for improving physical health status in adults and elderly Koreans. Our results suggest that it might be more effective to set different goals in terms of exercise frequency, intensity, time, and duration when devising physical activity plans for adults and the elderly. Furthermore, we need to develop various intervention programs (e.g., focused fun, competition, and social activity), rather than stress exercise types, to promote physical activity among adolescents.

## Figures and Tables

**Table 1:** Participant characteristics

	Variables	Adolescents (under 18 years)	Adults (19–64 years)	Elderly (over 65 years)
	Age (years)	15.67± 2.04	41.61 ± 12.88	71.75 ± 4.30

Sex	Male	586 (51.2)	3,247 (50.2)	646 (46.7)
	Female	558 (48.8)	3,227 (49.8)	736 (53.3)

Physical health status	So-so and below	90 (7.9)	1,300 (20.1)	851 (61.6)
	Quite and very healthy	1,054 (92.1)	5,174 (79.9)	531 (38.4)

Exercise frequency	None	689 (60.2)	3,169 (48.9)	767 (55.5)
	Two or three times per month	32 (2.8)	379 (5.9)	19 (1.4)
	Once per week	115 (10.1)	560 (8.6)	61 (4.4)
	Two times per week	106 (9.3)	486 (7.5)	64 (4.6)
	Three times per week	80 (7.0)	713 (11.0)	111 (8.0)
	4 times per week	18 (1.6)	310 (4.8)	68 (4.9)
	5 times per week	66 (5.8)	439 (6.8)	89 (6.4)
	6 times per week	20 (1.7)	143 (2.2)	56 (4.1)
	Every day	18 (1.6)	275 (4.2)	147 (10.6)

Exercise time (min)	None	689 (60.2)	3,169 (48.9)	767 (55.5)
	<59	85 (7.4)	387 (6.0)	138 (10.0)
	60–119	232 (20.3)	1,850 (28.6)	354 (25.6)
	120–179	110 (9.6)	698 (10.8)	80 (5.8)
	>180	28 (2.4)	370 (5.7)	43 (3.1)

Exercise duration (months)	None	689 (60.2)	3,169 (48.9)	767 (55.5)
	<23	233 (20.4)	838 (12.9)	68 (4.9)
	24–47	157 (13.7)	1,154 (17.8)	169 (12.2)
	48–71	39 (3.4)	664 (10.3)	143 (10.3)
	72–95	19 (1.7)	134 (2.1)	38 (2.7)
	>96	7 (0.6)	515 (8.0)	197 (14.3)

Exercise intensity	None	689 (60.2)	3,169 (48.9)	767 (55.5)
	Low	29 (2.5)	533 (8.2)	245 (17.7)
	Medium	295 (25.8)	2,294 (35.4)	353 (25.5)
	Vigorous	131 (11.5)	478 (7.4)	17 (1.2)

Data are expressed as means ± standard deviations or n (%)

**Table 2:** Results of the multivariate logistic regression analyses for physical health status in relation to the frequency, intensity, time, and duration of exercise in adolescents, adults, and the elderly Koreans (N = 9,000)

Physical health state		So-so and below Vs. Quite and very healthy								
		Adolescents (under 18 years)			Adults (19–64 years)			Elderly (over 65 years)		
		OR	95% CI	p	OR	95% CI	p	OR	95% CI	p
Exercise frequency	None	Ref.			Ref.		Ref.			
	Two or three times per month	0.657	0.221–1.955	0.450	1.079	0.819–1.422	0.589	2.242	0.888–5.661	0.087
	Once per week	1.694	0.705–4.071	0.238	1.692	1.310–2.187	<0.001^***^	1.473	0.855–2.538	0.163
	Two times per week	1.173	0.541–2.547	0.686	1.306	1.017–1.679	0.037^*^	2.688	1.588–4.550	<0.001^***^
	Three times per week	1.830	0.641–5.230	0.259	1.279	1.035–1.580	0.023^*^	1.772	1.173–2.675	0.007^**^
	4 times per week	<0.001	<0.001–<0.001	0.998	1.727	1.244–2.397	0.001^**^	1.746	1.043–2.922	0.034^*^
	5 times per week	2.976	0.701–12.633	0.139	1.445	1.107–1.887	0.007^**^	2.588	1.649–4.062	<0.001^***^
	6 times per week	<0.001	<0.001–<0.001	0.998	2.086	1.292–3.370	0.003^**^	3.212	1.837–5.617	<0.001^***^
	Every day	1.813	0.236–13.910	0.567	1.456	1.068–1.986	0.017^*^	2.331	1.619–3.357	<0.001^***^
Exercise Time (min)	None	Ref.			Ref.			Ref.		
	<59	4.157	0.994–17.386	0.051	0.833	0.650–1.067	0.149	1.518	1.036–2.224	0.032^*^
	60–119	1.862	0.950–3.650	0.070	1.488	1.277–1.733	<0.001^***^	2.173	1.670–2.827	<0.001^***^
	120–179	0.863	0.415–1.793	0.692	1.523	1.207–1.922	<0.001^***^	2.427	1.512–3.895	<0.001^***^
	Over 180	1.106	0.252–4.860	0.894	1.969	1.448–2.678	<0.001^***^	6.054	2.972–12.332	<0.001^***^
Exercise Duration (month)	None	Ref.			Ref.			Ref.		
	<23	2.384	1.154–4.926	0.019^*^	1.092	0.888–1.343	0.404	1.161	0.682–1.977	0.582
	24–47	1.192	0.599–2.374	0.617	1.436	1.199–1.721	<0.001^***^	1.813	1.282–2.565	0.001^**^
	48–71	0.789	0.267–2.336	0.669	1.359	1.095–1.686	0.005^**^	2.427	1.680–3.506	<0.001^***^
	72–95	1.582	0.205–12.208	0.660	2.385	1.440–3.952	0.001^**^	2.263	1.165–4.394	0.016^*^
	>96	<0.001	<0.001–<0.001	0.999	1.862	1.456–2.382	<0.001^***^	2.887	2.079–4.007	<0.001^***^
Exercise intensity	None	Ref.			Ref.			Ref.		
	Low	1.381	0.320–5.954	0.665	1.083	0.867–1.352	0.482	1.635	1.206–2.217	0.002^**^
	Medium	1.312	0.764–2.252	0.325	1.418	1.231–1.634	<0.001^***^	2.638	2.027–3.434	<0.001^***^
	Vigorous	3.885	1.181–12.773	0.025^*^	2.240	1.632–3.074	<0.001^***^	2.214	0.833–5.884	0.111

OR: Odds Ratio; CI: Confidence Interval

* p < 0.05,

** p < 0.01,

*** p < 0.001; tested by multivariable logistic regression analysis after adjusting for covariate variables such as sex and age

## References

[b1-jhk-45-261] Aaltonen S, Leskinen T, Morris T, Alen M, Kaprio J, Liukkonen J, Kujala U (2012). Motives for and barriers to physical activity in twin pairs discordant for leisure time physical activity for 30 years. Int J Sports Med.

[b2-jhk-45-261] Apor P (2011). Measure of fitness and physical activity related to cardiovascular diseases and death. Orv Hetil.

[b3-jhk-45-261] Bauman AE, Sallis JF, Dzewaltowski DA, Owen N (2002). Toward a better understanding of the influences on physical activity: the role of determinants, correlates, causal variables, mediators, moderators, and confounders. Am J Prev Med.

[b4-jhk-45-261] Brodersen NH, Steptoe A, Boniface DR, Wardle J (2007). Trends in physical activity and sedentary behaviour in adolescence: ethnic and socioeconomic differences. Br J Sports Med.

[b5-jhk-45-261] Christie D, Viner R (2005). Adolescent development. BMJ.

[b6-jhk-45-261] Colberg SR, Sigal RJ, Fernhall B, Regensteiner JG, Blissmer BJ, Rubin RR, Chasan-Taber L, Albright AL, Braun B, American College of Sports Medicine, American Diabetes Association (2010). Exercise and type 2 diabetes: the American College of Sports Medicine and the American Diabetes Association: joint position statement. Diabetes Care.

[b7-jhk-45-261] Dishman RK, Heath GW, Washburn R (2004). Physical activity epidemiology.

[b8-jhk-45-261] Giles-Cortia B, Donovan RJ (2002). The relative influence of individual, social and physical environment determinants of physical activity. Soc Sci Med.

[b9-jhk-45-261] Hagen KB, Dagfinrud H, Moe RH, Østerås N, Kjeken I, Grotle M, Smedslund G (2012). Exercise therapy for bone and muscle health: an overview of systematic reviews. BMC Med.

[b10-jhk-45-261] Korea Ministry of Culture, Sports and Tourism (2012). Korea Survey on Citizens’ Sports Participation. Korea Ministry of Culture, Sports and Tourism. (in Korean).

[b11-jhk-45-261] Penedo FJ, Dahn JR (2005). Exercise and well-being: a review of mental and physical health benefits associated with physical activity. Curr Opin Psychiatry.

[b12-jhk-45-261] Sandvik L, Erikssen J, Thaulow E, Erikssen G, Mundal R, Rodahl K (1993). Physical fitness as a predictor of mortality among healthy, middle aged Norwegian men. N Engl J Med.

[b13-jhk-45-261] Schmitz N, Kruse J, Kugler J (2004). The association between physical exercises and health-related quality of life in subjects with mental disorders: results from a cross-sectional survey. Prev Med.

[b14-jhk-45-261] Scott K, Edward T (2012). Exercise Physiology.

[b15-jhk-45-261] Van Der Horst K, Paw MJ, Twisk JW, Van Mechelen W (2007). A brief review on correlates of physical activity and sedentariness in youth. Med Sci Sports Exerc.

[b16-jhk-45-261] World Health Organization (2013a). Obesity and overweight.

[b17-jhk-45-261] World Health Organization (2013b). A global brief on hypertension: Silent killer, global public health crisis.

